# F-Actin nucleated on chromosomes coordinates their capture by microtubules in oocyte meiosis

**DOI:** 10.1083/jcb.201802080

**Published:** 2018-08-06

**Authors:** Mariia Burdyniuk, Andrea Callegari, Masashi Mori, François Nédélec, Péter Lénárt

**Affiliations:** Cell Biology and Biophysics Unit, European Molecular Biology Laboratory, Heidelberg, Germany

## Abstract

Capture of each and every chromosome by spindle microtubules is essential to prevent chromosome loss and aneuploidy. In somatic cells, astral microtubules search and capture chromosomes forming lateral attachments to kinetochores. However, this mechanism alone is insufficient in large oocytes. We have previously shown that a contractile F-actin network is additionally required to collect chromosomes scattered in the 70-µm starfish oocyte nucleus. How this F-actin–driven mechanism is coordinated with microtubule capture remained unknown. Here, we show that after nuclear envelope breakdown Arp2/3-nucleated F-actin “patches” form around chromosomes in a Ran-GTP–dependent manner, and we propose that these structures sterically block kinetochore–microtubule attachments. Once F-actin–driven chromosome transport is complete, coordinated disassembly of F-actin patches allows synchronous capture by microtubules. Our observations indicate that this coordination is necessary because early capture of chromosomes by microtubules would interfere with F-actin–driven transport leading to chromosome loss and formation of aneuploid eggs.

## Abstract (precis)

An actin network moves chromosomes to the cortex of oocytes during asymmetric division. Burdyniuk et al. show that in starfish oocytes actin is nucleated around chromosomes in a RanGTP- and Arp2/3-dependent manner. These F-actin “patches” coordinate chromosome capture in the large oocyte by preventing the formation of premature kinetochore–microtubule attachments. ​

## Introduction

Capture of chromosomes by spindle microtubules is an early step of cell division essential for subsequent alignment and segregation of chromosomes by the spindle apparatus. Failure to capture even a single chromosome will delay mitotic progression and may result in aneuploidy in somatic cells, a source of carcinogenesis. Because in oocyte meiosis the spindle assembly checkpoint is weakened or absent, in egg cells failure to capture chromosomes ultimately leads to aneuploidy (Kolano et al., 2012; Shao et al., 2013). Aneuploid eggs develop to unviable or severely impaired embryos, which in humans is one of the most common causes of infertility and birth defects (Webster and Schuh, 2017).

Mitchison and Kirschner (Mitchison and Kirschner, 1984; Kirschner and Mitchison, 1986) recognized that “dynamic instability,” the rapid growth and shrinkage of microtubules, is an effective means for centrosome-nucleated microtubules to explore the cellular space in their search for chromosomes. The proposed microtubule “search-and-capture” has since been validated in live cells (Hayden et al., 1990; Rieder and Alexander, 1990), and the molecular details of the initial attachments have also been understood (Tanaka, 2012). These so-called lateral attachments form between the kinetochore and the microtubule lattice and involve molecular motors, dynein in particular, which transport captured chromosomes poleward (Rieder and Alexander, 1990; Yang et al., 2007). Subsequently, these lateral attachments are replaced by end-on attachments to allow biorientation of chromosomes on the spindle (Shrestha and Draviam, 2013). Computer simulations recapitulated key features of search-and-capture, confirming that this mechanism is sufficient to reliably capture chromosomes in a typical, 30-µm, rounded somatic cell (Holy and Leibler, 1994; Wollman et al., 2005; Heald and Khodjakov, 2015).

Oocytes are much larger than somatic cells because they store cytoplasmic as well as nuclear constituents to support early embryonic development; hence, oocytes have not only a large cytoplasm but also a large nucleus, historically referred to as the germinal vesicle (Lénárt and Ellenberg, 2003). During meiosis, oocytes divide extremely asymmetrically to retain these stored components in the fertilizable egg. For this reason, across animal species, the meiotic spindle is small and located very eccentrically, anchored to the cell cortex to produce tiny polar bodies (Crowder et al., 2015).

The specific cellular geometry of oocytes challenges chromosome search-and-capture. Indeed, we have shown that in starfish oocytes the known microtubule-driven mechanisms are insufficient, and a contractile actin filament (F-actin) network is additionally required to transport chromosomes to the assembling microtubule spindle (Lénárt et al., 2005; Mori et al., 2011; Bun et al., 2018). The F-actin network forms in the nuclear space at nuclear envelope breakdown (NEBD) and transports embedded chromosomes to the microtubule asters located at a cortical position called the animal pole (AP). Thus, chromosome congression in starfish oocytes is a two-step process, whereby F-actin–dependent transport delivers chromosomes for capture by spindle microtubules. Whether and how chromosome capture is coordinated with F-actin–driven transport remained unknown.

Here, we tracked chromosomes in 3D at high spatiotemporal resolution in live oocytes to identify individual chromosome capture events. Our data indicate that capture of chromosomes by microtubules needs to be coordinated with F-actin–driven transport, because early capture events would interfere with F-actin–driven transport by causing a local collapse of the F-actin network. We show that this coordination is achieved by Arp2/3-nucleated F-actin patches surrounding chromosomes. These patches form in a Ran-GTP–dependent manner at NEBD and sterically prevent microtubule–kinetochore attachments for ∼5 min after NEBD. We integrate these results in a computational model and find that two-step chromosome congression in starfish oocytes is well explained by the classical search-and-capture model when we add the single feature of preventing chromosome capture during the initial F-actin–driven transport.

## Results

### Microtubule-capture events can be identified on high-resolution chromosome trajectories

**Figure 1.**
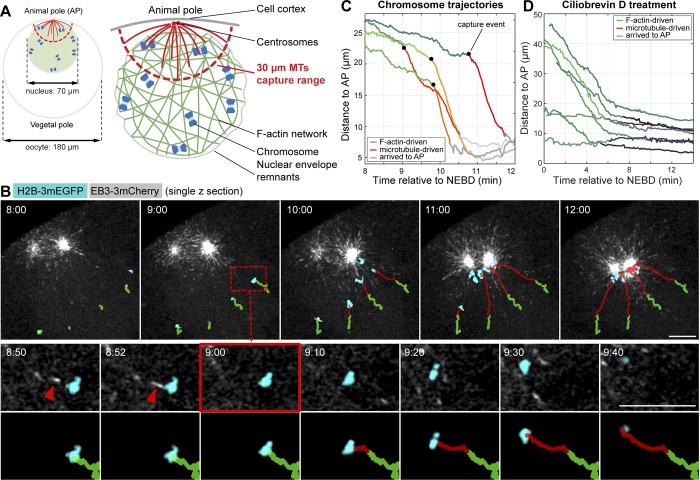
**After F-actin–driven congression, chromosomes form lateral attachments and are transported along microtubules to the spindle poles. (A)** Left: Scheme of an immature starfish oocyte with the nucleus anchored at the AP and with centrosomes nucleating astral microtubules. Right: Schematics of the nuclear region after NEBD. The F-actin network fills the nuclear region and, as it contracts, transports embedded chromosomes toward the AP. Chromosomes delivered within the capture range of astral microtubules are captured and transported on microtubules to the centrosomes at the AP. **(B)** Selected frames from a time series of single confocal sections through the nuclear region of an oocyte expressing EB3-3mCherry to visualize microtubule plus-tips (gray) and H2B-3mEGFP to label the chromosomes (cyan). See also Video 1. Chromosome trajectories are overlaid onto the images: green denotes actin-driven transport and red shows microtubule-driven transport. Lower panels: Zoom of the area marked with a dashed square, and the selected time points around microtubule capture are shown. Red arrowheads mark the contact between the microtubule and chromosome. Bars: (main images) 10 µm; (insets) 5 µm. **(C)** Plot of distance of chromosomes to the AP over time, calculated from the trajectories shown in B. Black dots mark capture events identified as the transition point between slow, F-actin–driven, and fast microtubule-driven transport. **(D)** Plot of chromosome distance to the AP over time in an oocyte treated with Ciliobrevin D to inhibit dynein (Fig. S1, G and H, for details). Time is given as minutes:seconds relative to NEBD.

Upon entry into meiosis, after the onset of NEBD, a contractile F-actin network forms in the 70-µm nucleus of starfish oocytes and transports chromosomes to the AP (Mori et al., 2011). Starfish oocytes do contain centrosomes, which form two microtubule asters at the AP (Borrego-Pinto et al., 2016a). We have shown earlier by tubulin immunofluorescence that these microtubules extend to 30–40 µm from the AP (Lénárt et al., 2005). Once the F-actin network transports chromosomes within this “capture range,” chromosomes are caught and are eventually incorporated into the first meiotic spindle forming at the AP (Lénárt et al., 2005; Fig. 1 A).

To visualize individual chromosome capture events in live oocytes, we imaged chromosomes and growing microtubule tips by acquiring single confocal sections at high spatiotemporal resolution, starting from NEBD until chromosomes are collected at the AP (Fig. 1 B and Video 1). We then automatically tracked chromosome motion (Mori et al., 2011; Monnier et al., 2012). By comparison of chromosome trajectories to microtubule dynamics, we could clearly identify individual events of capture: initially the F-actin network transported chromosomes at a lower speed and with the motion being less directed and more diffusive (Fig. 1, B and C, green trajectories). Then, shortly after (10–30 s) a visible direct contact to a microtubule, the chromosomes switched to a faster and directed motion (Fig. 1, B and C, red trajectories). Similar to these 2D recordings with microtubule tips and chromosomes colabeled, we were also able to unambiguously identify such capture events in the entire 3D nuclear volume by imaging chromosomes alone. Because of the bright labeling provided by H2B-3mEGFP, we were able to acquire volumes every 3 s, which was sufficient to resolve the transition point between the slow, F-actin–driven motion and fast transport along microtubules (Fig. S1, A–D; and see Materials and methods for details).

The speed of motion (9.22 ± 2.86 µm/min) after the switch matched well the speed expected for dynein-driven transport after establishment of canonical lateral kinetochore–microtubule attachments (Yang et al., 2007; Barisic et al., 2014). Consistently, transport was abolished by the dynein small-molecule inhibitor, Ciliobrevin D (Fig. 1 D and Fig. S1, G and H).

Together, these observations establish that chromosome capture events can be reliably identified by analysis of chromosome trajectories as the transition point between the slow and more diffusive F-actin–driven and the fast and directed dynein-driven transport.

### Chromosome capture is coordinated by an F-actin–dependent mechanism

Being able to identify capture events allowed us to next ask how F-actin–driven transport and microtubule capture are coordinated during chromosome congression. To this end, we compared chromosome capture in untreated control oocytes and oocytes in which F-actin–driven transport was abolished.

**Figure 2.**
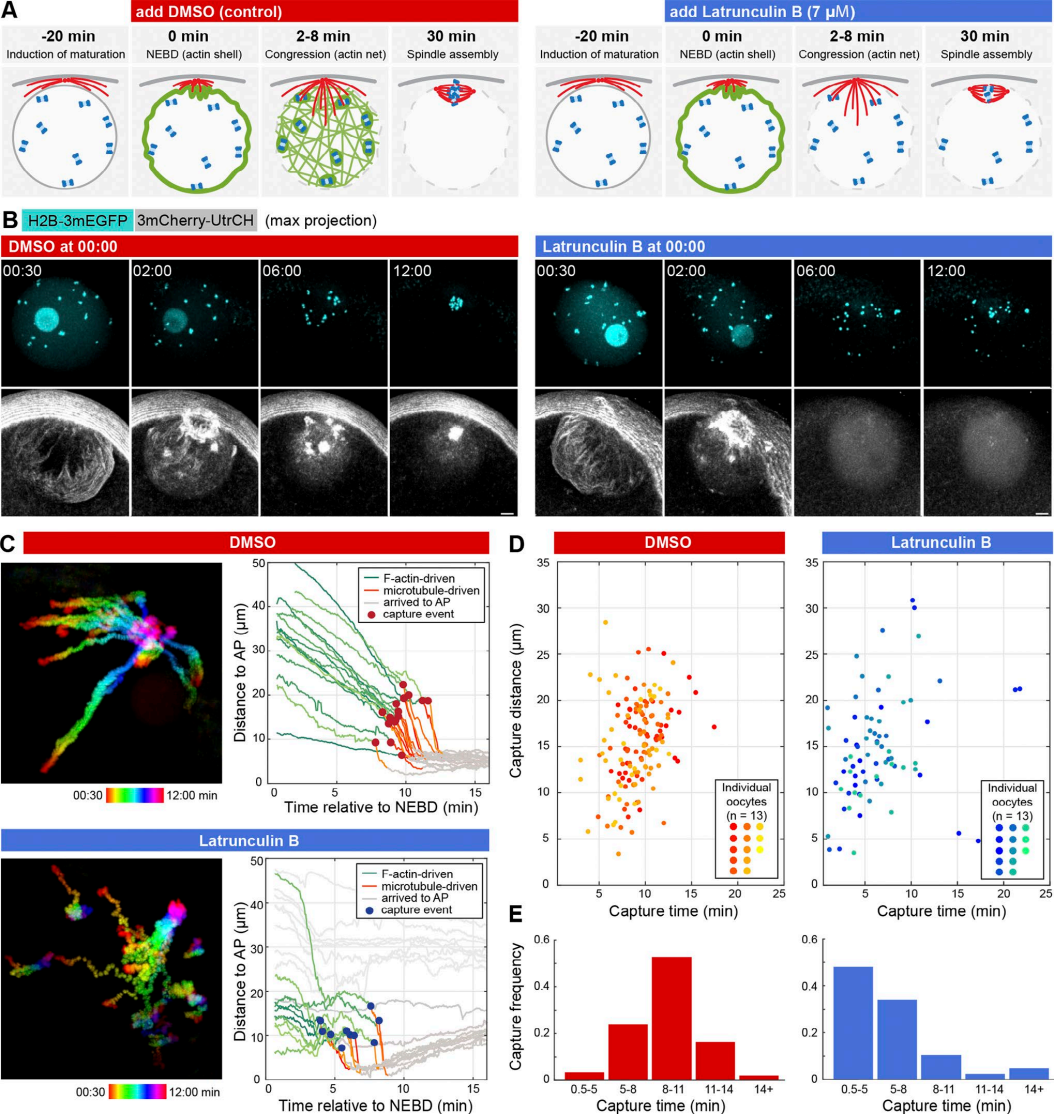
**An F-actin–dependent mechanism delays chromosome capture. (A)** Schematics of the experimental protocol for acute depolymerization of F-actin. **(B)** Selected maximum-intensity z-projections from a 3D confocal time series though the oocyte’s nuclear region during chromosome congression. Chromosomes (H2B-3mEGFP) are in cyan and F-actin (3mCherry-UtrCH) in gray. Bars, 10 µm. See also Video 2. **(C)** Left: Pseudo-colored time projection of a 3D confocal time series of chromosome congression (labeled with H2B-3mEGFP) in control or Latrunculin B–treated oocytes. See also Video 3. Right: Plot of chromosome distance to the AP over time, for the control and Latrunculin B–treated oocytes shown on the left. **(D)** Chromosome capture events identified for 13 pairs of control and Latrunculin B–treated oocytes (plotted in a different color for each oocyte). **(E)** Histograms of the data shown in D. Time is given relative to NEBD for all panels.

To inhibit F-actin–driven transport, we developed a protocol to acutely depolymerize F-actin after NEBD, because as we had shown earlier, NEBD in starfish oocytes requires F-actin (Mori et al., 2014; Fig. 2 A). Therefore, Latrunculin B (or equal amount of DMSO for controls) was added to the oocytes immediately after NEBD, allowing the formation of the “F-actin shell” required for the rupture of nuclear membranes (Fig. 2 B, 00:30). Latrunculin B then rapidly (in 2–3 min) disrupted all F-actin structures and abolished F-actin–driven chromosome transport, resulting in a massive loss of chromosomes distal to the AP (Fig. 2 B and Video 2). The affected F-actin structures included the cell cortex, the F-actin network in the nuclear region, as well as dense patches of F-actin, which were previously observed surrounding chromosomes (Fig. 2 B; Lénárt et al., 2005).

We next combined this protocol with high-resolution tracking of chromosome motion as above. In control oocytes, trajectories showed the two clearly distinguishable phases of F-actin– and microtubule-driven transport (Fig. 2 C and Video 3). The transition between the two phases, i.e., the capture events, occurred rather synchronously (9.21 ± 2.36 min after NEBD) showing a slight distance dependence (Fig. 2, C and D). Upon acute F-actin depolymerization, distal chromosomes outside of the microtubule-capture range were lost, as expected (Fig. 2, B and C; Mori et al., 2011). However, to our surprise, chromosomes initially positioned within the microtubule-capture range were efficiently captured by microtubules, and capture of these chromosomes occurred earlier than in controls (6.16 ± 3.92 min after NEBD; Fig. 2, B–D). This difference is clearly visualized on scatter plots accumulating data from multiple oocytes and histograms of capture events: the majority of captures occur between 8 and 11 min after NEBD in DMSO controls, in contrast to the peak between 0.5 and 5 min for Latrunculin B–treated oocytes (Fig. 2, D and E).

Thus, in control oocytes capture of chromosomes is prevented for the ∼5 min after NEBD. In contrast, when F-actin is depolymerized, capture starts immediately after NEBD (Fig. 2 D). This implies that besides chromosome transport, F-actin plays an additional role in coordinating spindle assembly by delaying capture of chromosomes by microtubules.

### The F-actin network is disrupted by chromosomes transported along microtubules

To explore the potential function and mechanism underlying the F-actin–dependent delay of chromosome capture, we first characterized the F-actin network that forms in the nuclear region at NEBD and transports chromosomes (Lénárt et al., 2005; Mori et al., 2014). We hypothesized that the function of this delay may be that capture by microtubules interferes with transport by the F-actin network, and for this reason capture by microtubules needs to be delayed until F-actin–driven transport is complete. As for the underlying mechanisms, we considered two scenarios: (1) coordination may be achieved by the F-actin network hindering access of microtubules to chromosomes and (2) alternatively, microtubule attachments could form in an uninhibited manner, but the F-actin network might physically entrap chromosomes to prevent their poleward transport.

**Figure 3.**
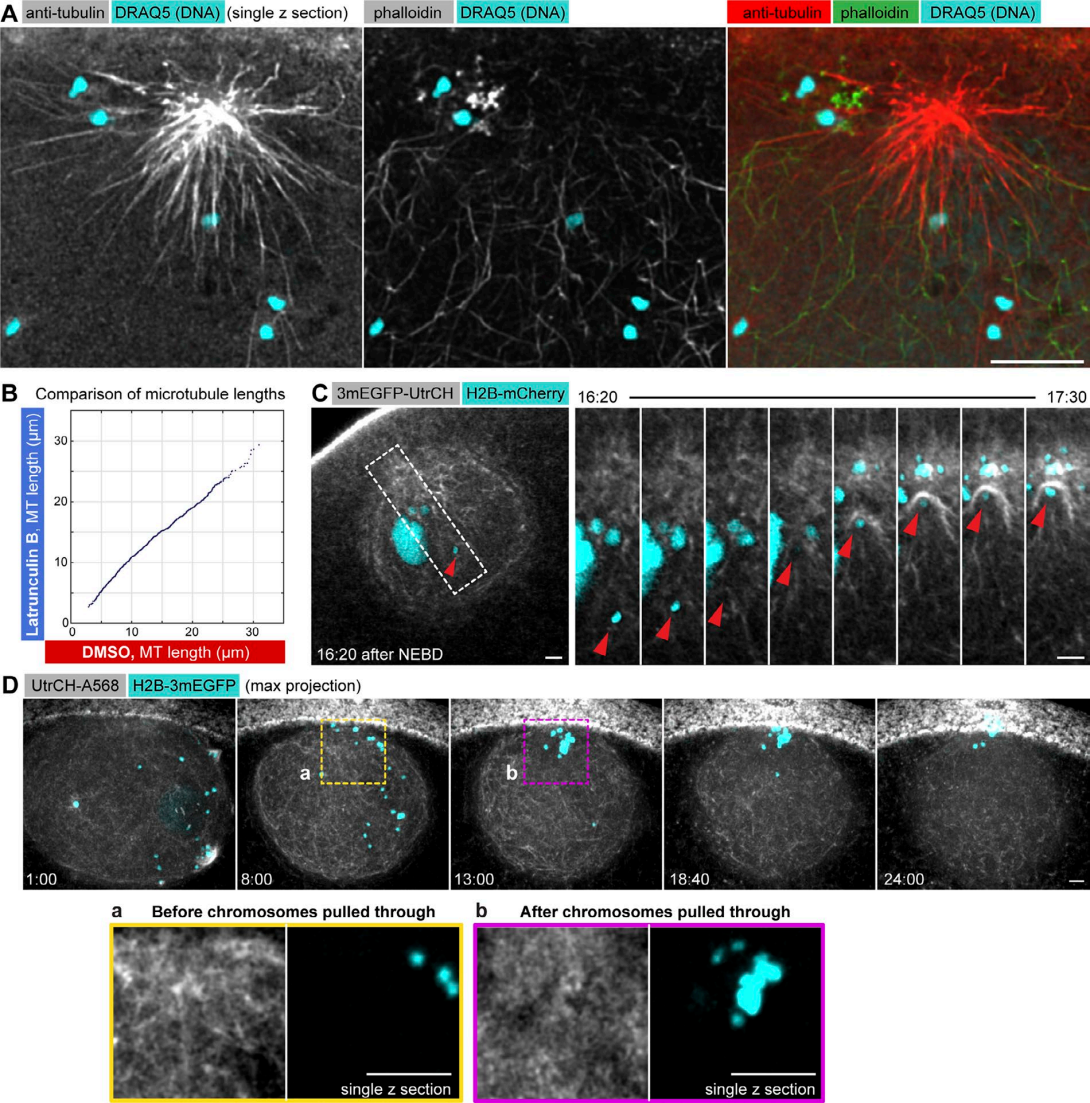
**The F-actin network does not prevent chromosome capture and transport by microtubules, but transport along microtubules interferes with F-actin network integrity. (A)** A single selected slice from a deconvolved confocal stack of an oocyte fixed 5 min after NEBD and stained for tubulin (red), F-actin (green), and chromosomes by using Draq5 (cyan). **(B)** Microtubule length distribution measured in control and Latrunculin B–treated oocytes and plotted against each other. **(C)** Selected single confocal sections acquired over time showing the nuclear area of an oocyte expressing 3mEGFP-UtrCH (gray) and H2B-mCherry (cyan). See also Video 4. Right: Zoom-in on the region marked by a dashed rectangle of a chromosome transported along a microtubule (red arrowhead) causing local collapse of the F-actin network. **(D)** Maximum-intensity z-projections of a 3D confocal time series through the nuclear region of an oocyte expressing H2B-mCherry (cyan) and injected with UtrCH–Alexa Fluor 568 (gray). Single z-slice zooms of the regions marked by dashed rectangles are shown below visualizing the disruption of the F-actin network where chromosomes are pulled through. Bars: (main images) 10 µm; (C, inset) 5 µm. Time is given as minutes:seconds relative to NEBD for all panels.

To test these possibilities, we first fixed and costained oocytes for F-actin and microtubules and imaged these at high resolution in 3D. We clearly visualized astral microtubules penetrating the F-actin network (Fig. 3 A). Further, we could not detect inhomogeneities in the microtubule density, suggesting that microtubules can grow just as freely into the F-actin network as in the cytoplasm (Fig. 3 A). Additionally, we monitored microtubule dynamics in live oocytes treated with either Latrunculin B or DMSO using the protocol above (Fig. 2 A). Quantification of microtubule lengths showed no significant difference between Latrunculin B– and DMSO-treated oocytes (Figs. 3 B and S2 A). Based on these results, we conclude that the F-actin network does not present an obstacle to microtubule growth, and therefore the F-actin network, per se, does not restrict access of microtubules to chromosomes.

We then tested whether the F-actin network retains chromosomes by direct binding between chromatin and actin filaments, thereby preventing their transport along microtubules. Therefore, we generated chromatin fragments by treating oocytes with Zeocin (Fig. S2 B). Small fragments of ∼0.5 µm were sieved through the F-actin network and diffused freely in the nucleoplasm, whereas large fragments were trapped and transported by the network (Fig. S2 B), remarkably similar to the behavior of inert beads of comparable sizes, as shown earlier (Mori et al., 2011). This suggests that chromatin does not directly interact with filaments of the F-actin network.

Even if chromatin did not directly bind F-actin, chromosomes are sufficiently large so that the F-actin network may hinder transport along microtubules by physically entrapping them. To test this, we recorded videos with chromosomes and F-actin colabeled. We could clearly visualize chromosomes being captured and pulled through the still-intact F-actin network, dragging F-actin bundles along (Fig. 3 C and Video 4). Capture and transport occurred efficiently even when filaments were strongly stabilized by phalloidin (Fig. S2 C). These results indicate that the F-actin network is not able to resist chromosomes transported on microtubules. Importantly, the videos additionally revealed that such pulling through of chromosomes by microtubules causes a local collapse and disruption to the F-actin network, “clearing out” the network where chromosomes pass (Fig. 3 D).

Together, these data show that transport along microtubules leads to local disruption of the F-actin network by chromosomes being pulled through, confirming our hypothesis that capture by microtubules needs to be delayed for F-actin–driven transport to function effectively. However, we show that the F-actin network does not present an obstacle for microtubules to grow toward, to capture, or to transport chromosomes poleward; thus the network itself is unlikely to be the F-actin structure responsible for mediating this delay in chromosome capture.

### Disassembly of F-actin patches coordinates capture by microtubules

As the F-actin network is unlikely to be coordinating chromosome capture, we next focused on the other prominent F-actin structure present in the nuclear region during this critical time period: F-actin patches that have been observed to form around chromosomes but otherwise remained poorly characterized (Lénárt et al., 2005).

**Figure 4.**
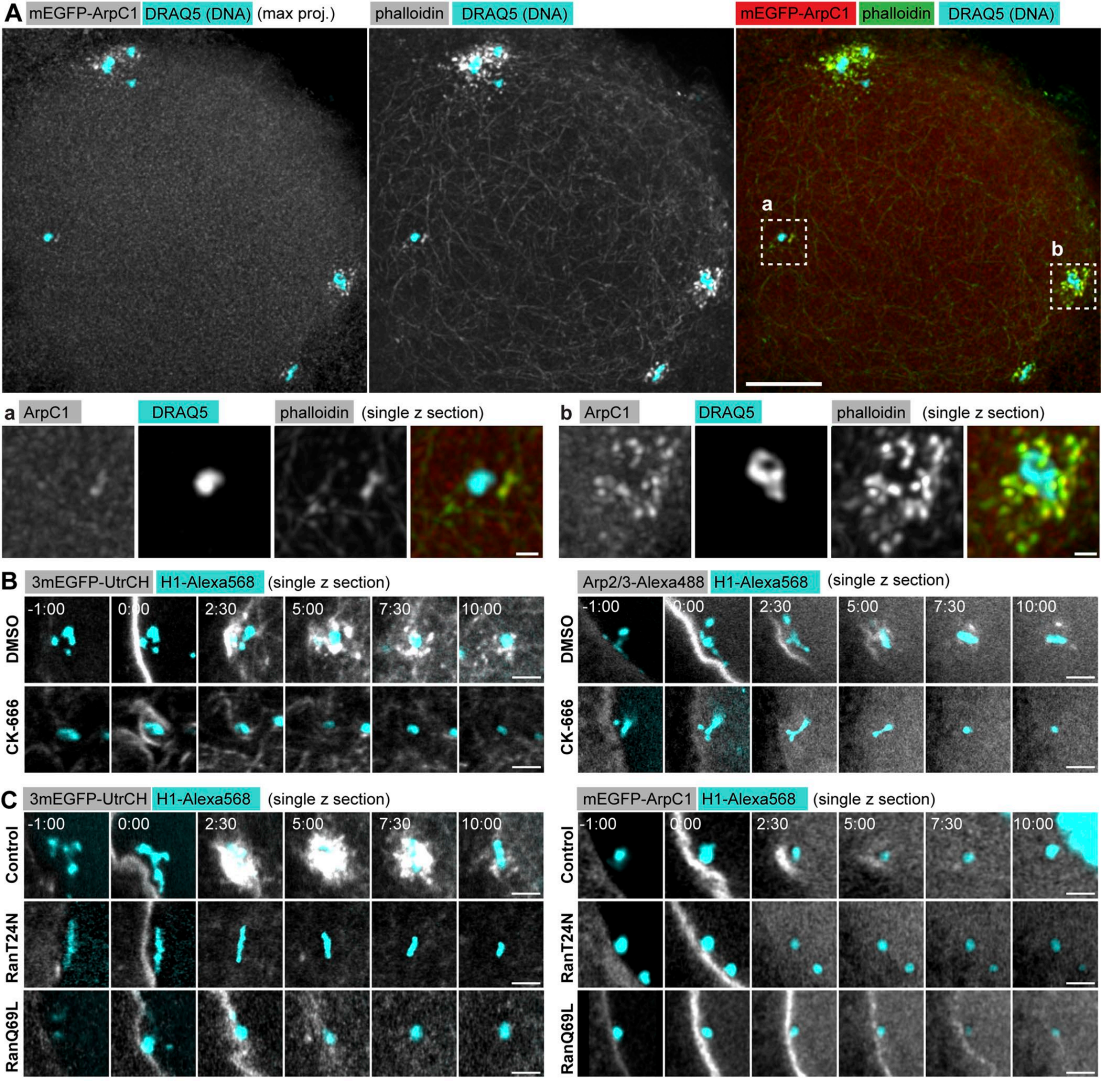
**F-actin patches are nucleated on chromosomes by the Arp2/3 complex in a Ran-dependent manner. (A)** Maximum projection of selected z-sections from a confocal z-stack of an oocyte expressing mEGFP-ArpC1 fixed 5 min after NEBD and immunostained. Anti-GFP antibody was used to enhance mEGFP-ArpC1, Phallodin-A568 to stain F-actin, and Draq5 for DNA. Below: Selected single z-slices zooming in on F-actin patches marked by dashed rectangles on the overview. Bars: (top images) 10 µm; (bottom images) 1 µm. **(B)** Single confocal slices selected from a time series of an oocyte injected with H1–Alexa Fluor 568 (cyan) and expressing either 3mEGFP-UtrCH (gray) to label F-actin or injected with Arp2/3–Alexa Fluor 488 protein (gray) to visualize the Arp2/3 complex. A region around a selected chromosome is shown. Oocytes were treated with CK-666 or with equal amount of DMSO 1 h before maturation. **(C)** Single confocal slices selected from a time series of an oocyte injected with H1–Alexa Fluor 568 (cyan) and either 3mEGFP-UtrCH mRNA to visualize F-actin or Arp2/3–Alexa Fluor 488 protein to visualize the Arp2/3 complex. A region around a selected chromosome is shown. Oocytes were injected with RanT24N or RanQ69L protein or equal amount of buffer as control. Time is given as minutes:seconds relative to NEBD. Bars, 5 µm.

We first characterized their morphology and the mechanisms of nucleation. F-actin patches appear to be composed of spots of dense F-actin, reminiscent of endocytic sites or other Arp2/3-nucleated structures (Kaksonen et al., 2003), clearly distinct from the F-actin network composed of long filament bundles (Fig. 4 A). We detected patches of variable size and intensity on chromosomes, with peripheral chromosomes in contact with nuclear envelope membranes being surrounded by much larger and brighter patches, as compared with those located deeper in the nuclear volume (Fig. 4 A).

Consistent with their morphology, F-actin patches were specifically labeled by mEGFP-ArpC1, a subunit of the Arp2/3 nucleator complex (Fig. 4 A). Indeed, the small-molecule Arp2/3 inhibitor, CK-666, blocked recruitment of Arp2/3 and effectively prevented the formation of F-actin patches, leaving the F-actin network largely intact (Fig. 4 B; Mori et al., 2014). We thus conclude that the F-actin patches are nucleated by the Arp2/3 complex, whereas filaments of the F-actin network are polymerized by other factors, likely formins (Bun et al., 2018). We have shown previously that the F-actin patches are nucleated around DNA-coated beads (Lénárt et al., 2005). This further suggested the involvement of the small GTPase, Ran, possibly related to Ran- and Arp2/3-mediated actin nucleation reported in mouse oocytes (Deng et al., 2007). To test this hypothesis, we injected a large amount of RanT24N, a mutated version of Ran locked predominantly in its inactive, GDP-bound form and RanQ69L, defective in GTP hydrolysis and thus locked in its active form (Dasso et al., 1994; Görlich et al., 1996). RanT24N injection abolished F-actin patches, preventing recruitment of ArpC1 as well as actin nucleation (Fig. 4 C). RanQ69L also prevented F-actin patch formation locally on chromosomes and additionally caused a global recruitment of Arp2/3 and actin nucleation all along the nuclear region (Fig. 4 C). These data indicate that Arp2/3 recruitment and F-actin patch nucleation require locally produced Ran-GTP on chromatin.

**Figure 5.**
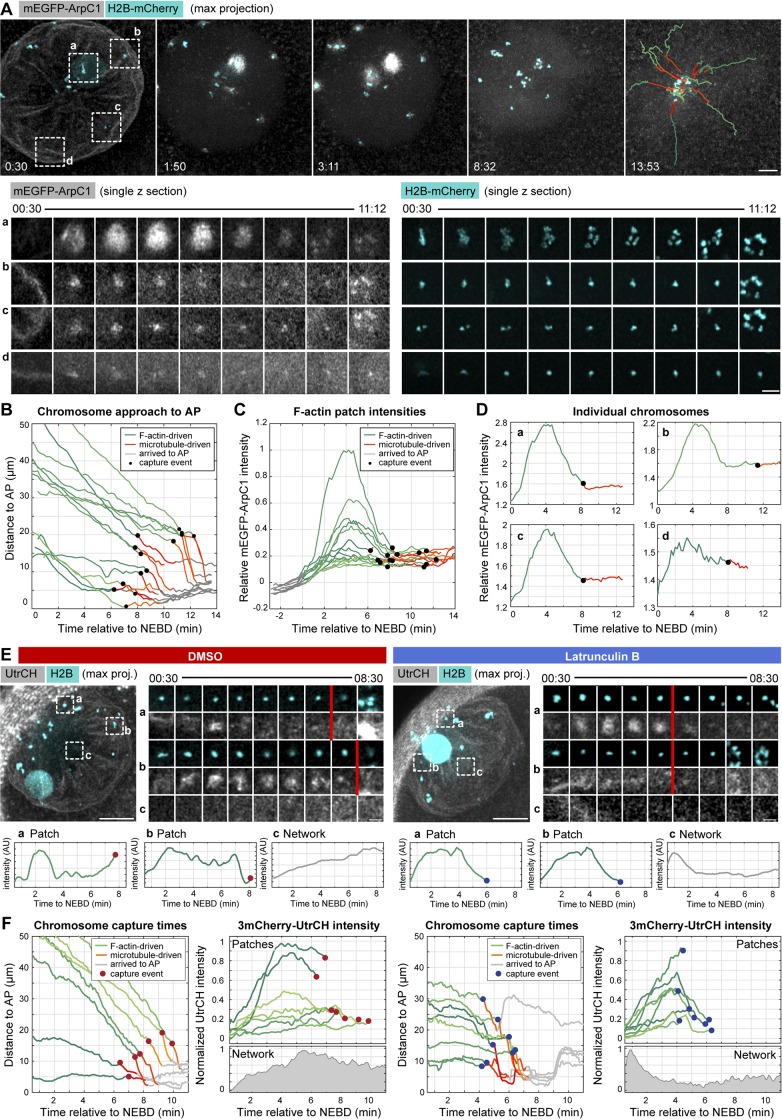
**Disassembly kinetics of F-actin patches are tightly correlated with chromosome capture by microtubules. (A)** Maximum-intensity z-projection of selected time points from a time series of the nuclear area of an oocyte expressing mEGFP-ArpC1 to label the Arp2/3 complex (gray), and H2B-mCherry to label chromosomes (cyan). Last frame: Chromosome tracks overlaid, color-coded as below. See also Video 5. Below: Single confocal slices of selected chromosomes marked by dashed rectangles on the overview. Bars: (top) 10 µm; (bottom) 5 µm. Time is given as minutes:seconds relative to NEBD. **(B)** Plot of chromosome distance to the AP over time, calculated from the trajectories shown in A. Trajectories are color-coded for actin- (green) and microtubule (red)-driven transport phases and arrival at the spindle (gray). Chromosome capture events are represented by black dots. **(C)** Normalized mEGFP-ArpC1 intensity profile for each chromosome tracked in B. Intensity is calculated in a 5-µm diameter sphere around the chromosome’s center of mass and normalized to the background level before NEBD onset (in gray). Plots are color-coded as in B. **(D)** Individual plots for chromosomes shown in A. **(E)** Oocytes expressing 3mCherry-UtrCH to label F-actin (gray) and H2B-3mEGFP to label chromosomes (cyan) were then treated with DMSO or Latrunculin B, respectively (left and right panels). Left side: Maximum z-projection of the first time point (00:30). Right side: Single confocal slices of selected chromosomes and an area sampling the F-actin network as marked by dashed rectangles on the overview. Below: Individual plots of mean intensities for chromosome patches (a and b) or network (c) shown above. Red bars indicate the chromosome capture event. Bars: (main images) 20 µm; (smaller images) 5 µm. Time is given as minutes:seconds relative to NEBD. **(F)** Plot of chromosome distance to the AP over time for the oocytes shown in E. Top right: Normalized 3mCherry-UtrCH intensity profiles for the 5-µm-diameter sphere surrounding each chromosome. Bottom right: Normalized mean F-actin network intensity profile.

Next, we quantified the assembly and disassembly kinetics of F-actin patches and correlated this with chromosome capture. To this end, we colabeled chromosomes and patches using mEGFP-ArpC1 as a marker and imaged these in 3D at high resolution in live oocytes (Fig. 5 A and Video 5). We then tracked chromosomes as above and, using chromosome coordinates as reference points, quantified the total patch intensity in a 2.5-µm-radius sphere around every chromosome for each time point (Fig. 5 C). These measurements showed that F-actin patches assemble within 1–2 min after NEBD on all chromosomes (with peripheral chromosomes followed by those deeper in the nucleus). The intensity of the patches peaked at around 5 min, followed by disassembly, with mEGFP-ArpC1 intensities dropping to background levels ∼8 min after NEBD (Fig. 5 C).

The disassembly kinetics of F-actin patches was remarkably coordinated and largely independent of initial patch size and intensity (Fig. 5, A and D). Strikingly, the kinetics of F-actin patch disassembly matched well the time of chromosome capture by microtubules (Fig. 5 B). The first capture events were detected 6–8 min after NEBD, when the first patches disassemble, and most chromosomes were captured very soon after F-actin patch disassembly, 8–11 min after NEBD.

We obtained similar results by quantifying F-actin around chromosomes labeled by 3mCherry–Utrophin CH domain (UtrCH), although the contrast was lower because 3mCherry-UtrCH also labels the F-actin network, unlike mEGFP-ArpC1, which is specific to patches (Fig. 5 E). However, this allowed us to simultaneously monitor F-actin network dynamics in these experiments. Comparisons of temporal dynamics of patch and network intensities reveal that, similar to mEGFP-ArpC1 above, patch disassembly correlates with chromosome capture. To the contrary, the F-actin network persists several minutes after chromosome capture is completed (Fig. 5, E and F). To test whether this correlation is maintained under perturbations, we treated oocytes with Latrunculin B to acutely depolymerize F-actin as above. Quantifications confirmed the premature disassembly of F-actin patches at ∼4 min after NEBD that was followed by chromosome capture events correlated with the decline in patch intensities (Fig. 5, E and F; and Fig. S3). In contrast, the F-actin network disassembled 2–3 min before patches, much earlier than chromosome capture occurred. Thus, in both unperturbed and Latrunculin B–treated oocytes, chromosome capture events are correlated with patch disassembly but not with network assembly/disassembly dynamics.

Collectively, patches are F-actin structures distinct from the network and nucleated by the Arp2/3 complex on chromosomes in a Ran-GTP–dependent manner. Quantitative analysis of their disassembly kinetics revealed a tight correlation with chromosome capture events, strongly suggesting that F-actin patches are responsible for coordinating chromosome capture. To the contrary, the disassembly kinetics of the network, the other prominent F-actin structure present in the nuclear region, is not correlated with capture events, as it persists long after chromosome capture is completed, indicating that the network is not involved in coordinating capture events.

### Search-and-capture extended by an early block recapitulates chromosome capture dynamics

**Figure 6.**
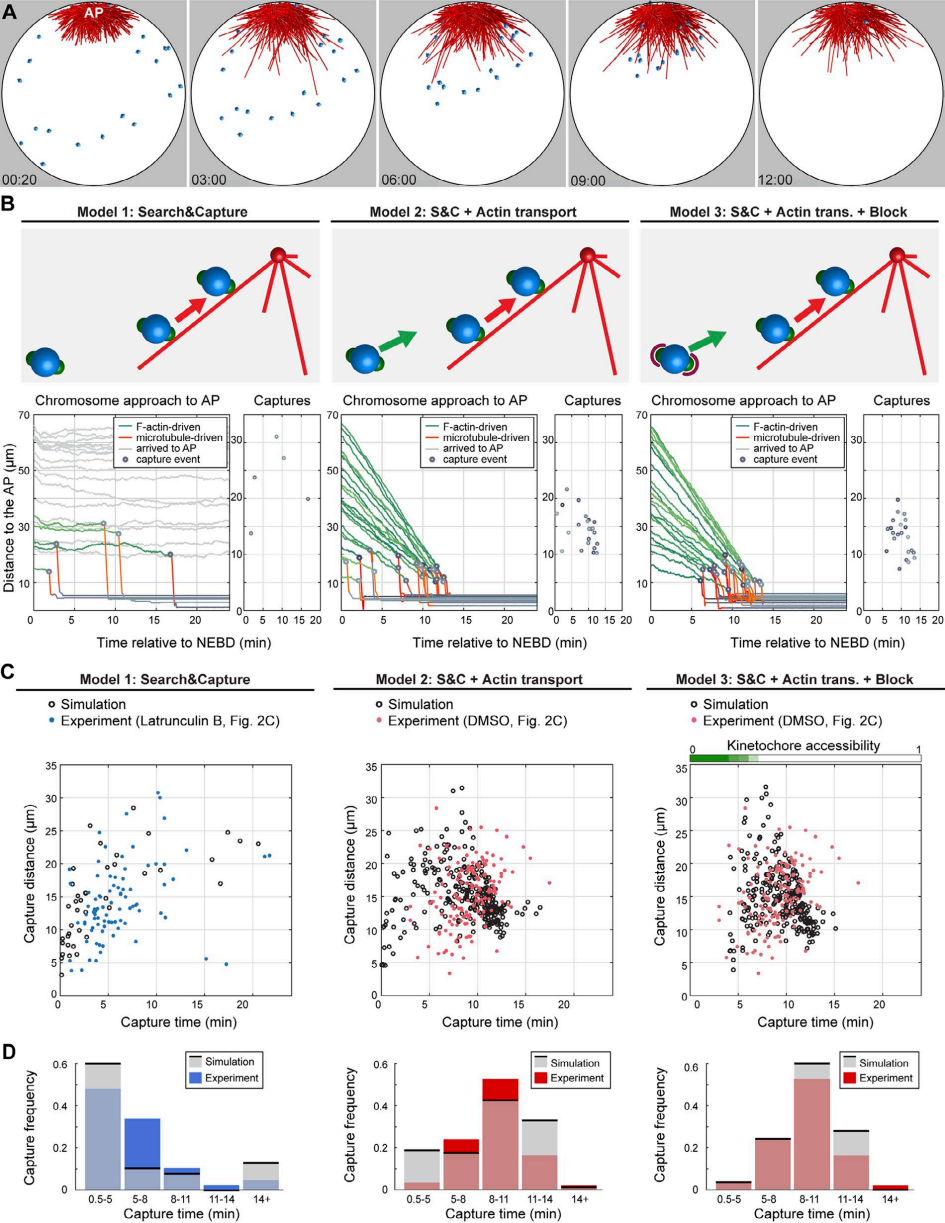
**Search-and-capture expanded by F-actin–driven transport and block of early capture explains chromosome capture dynamics. (A)** Renderings of 3D Cytosim simulations of two-staged chromosome congression. Microtubules are in red, chromosomes are blue spheres with green kinetochores. Time starts at NEBD. See also Video 6. **(B)** Schematics of the different models and corresponding plots of chromosome distance to the AP over time and capture events. Trajectories are color-coded for actin- (green) and microtubule (red)-driven transport phases and arrival at the spindle (gray). Chromosome capture events are represented by gray dots. **(C)** Comparison of simulated and experimental chromosome capture dynamics (observed in oocytes, treated with Latrunculin B or DMSO; Fig. 2). Data from 13 oocytes are shown for both simulations and experiments. **(D)** Plots of individual experimental and simulated capture events and histograms of the same data. S&C, search and capture; trans., transport.

Finally, we integrated our observations in a computational model using the Cytosim software (Nedelec and Foethke, 2007). Within the realistic 3D geometry of the starfish oocyte, microtubules were nucleated from centrosomes located at the AP with dynamics based on dynamic instability and parameters estimated experimentally (Fig. 6 A; Fig. S4, A–E; and Video 6; Mitchison and Kirschner, 1984; Wollman et al., 2005; Magidson et al., 2015).

We first simulated the classic search-and-capture, without F-actin driven transport. This model very closely recapitulated the capture kinetics observed in Latrunculin B–treated oocytes: distal chromosomes outside of the capture range (∼30 µm) were lost, whereas chromosomes within the capture range were captured one after the other by random search (Fig. 6, B–D, model 1). For chromosomes located close to the centrosomes, capture started immediately after NEBD. In the second model, we included a force field simulating the F-actin network that transports chromosomes toward the AP (Fig. 6, B–D, model 2; and Fig. S4 C). In this scenario, all 22 chromosomes were successfully captured by microtubules within ∼15 min, with similar kinetics to experiments. However, unlike in experiments, the first capture events occurred immediately after NEBD. Therefore, in the third model we introduced an additional feature to simulate F-actin patches. We set the binding rate of kinetochores to microtubules for the first 4 min after NEBD to 0 (no binding) and gradually increased it to 10, then 30, 70, and finally 100%, every minute, 4–8 min after NEBD, matching the disassembly kinetics of patches. Simulations with this feature added faithfully recapitulated chromosome capture dynamics observed experimentally (Fig. 6, B–D, model 3).

Thus, simulations show that the classical search-and-capture model is in principle able to explain chromosome capture in starfish oocytes. There are only two additional, F-actin-dependent features to be added: (1) transport of distal chromosomes by the F-actin network and (2) F-actin patches that delay capture of chromosomes until the transport by the F-actin network is complete, thereby ensuring that early capture events do not interfere with transport by the F-actin network.

## Discussion

Collectively, our results evidence a novel mechanism to coordinate chromosome capture that is mediated by F-actin patches nucleated on chromatin by the Arp2/3 complex in a Ran-GTP–dependent manner. We propose that these F-actin patches sterically block microtubule–kinetochore attachments until their synchronous disassembly. We show that in starfish oocytes, in which chromosomes are first transported by an F-actin network and then by microtubules, this coordination is necessary to ensure that F-actin–driven transport is complete before capture by microtubules. Our observations suggest that without this coordination early captured chromosomes would locally collapse the F-actin meshwork and thereby interfere with F-actin–driven transport of distally located chromosomes, leading to chromosome loss.

In terms of the underlying molecular mechanism, it is interesting to note the similarity to mouse oocytes, in which Arp2/3-nucleated and Arp2/3-independent, formin-nucleated F-actin networks coexist in the cytoplasm, each of them having separate, essential functions (Almonacid et al., 2014). Similarly, in starfish oocytes the F-actin network assembles in the nuclear region at NEBD in an Arp2/3-independent manner and functions to transport chromosomes. Simultaneously, Arp2/3 nucleates patches on chromosomes to delay capture by microtubules. In the future, it will be critical to develop assays in which one or the other F-actin structure can be selectively perturbed. Unfortunately, most regulators, such as disassembly or stabilizing factors, act on both, and inhibiting the specific nucleators is further complicated by the fact that these have multiple functions. In particular, Arp2/3 is essential for NEBD (Mori et al., 2014) just minutes before F-actin patches form. Therefore development of tools that allow acute perturbations, such as photoactivable small-molecule inhibitors or optogenetic reagents, will be necessary.

Another intriguing similarity between mouse and starfish oocytes is that in the mouse chromatin also induces formation of the so called “actin cap,” an Arp2/3-nucleated thickening of the cell cortex in a Ran-GTP–dependent manner (Deng et al., 2007). Ran-GTP has been proposed to act through Cdc42 and N-WASP in this case (Dehapiot et al., 2013). If this pathway was conserved, it could explain nucleation of actin on chromosomes in starfish oocytes analogous to Ran-mediated activation of spindle assembly factors (Hetzer et al., 2002). It will be very interesting in the future to explore the molecular details and conservation of the pathway leading to Ran-mediated Arp2/3 activation and subsequent inactivation resulting in synchronous disassembly of the F-actin patches. How it is controlled in space and time, how is it coupled to the cell cycle, and how it may contribute to coordinating chromosome capture in other systems remain exciting open questions.

An additional key question is how such dense F-actin structures prevent chromosome capture by microtubules. One possibility is a biochemical regulation mediated by molecules that interact with both cytoskeletal systems, many of which have been identified recently (Chesarone et al., 2010; Henty-Ridilla et al., 2016). However, we favor an alternative and not mutually exclusive mechanism, whereby the dense, branched F-actin network nucleated by Arp2/3 constitutes a physical barrier to microtubule growth (unlike formin-nucleated bundles that are expected to bend away and organize networks occupying a much smaller volume fraction). Indeed, in different contexts and cell types, particularly striking in axon growth cones, Arp2/3-nucleated F-actin networks have been observed to strictly exclude microtubules, resulting in sharp separation between the two zones (Lowery and Van Vactor, 2009). It will be very exciting to explore the physiological functions exclusion of microtubules by branched F-actin networks may have.

There are number of common features of oocyte meiosis shared across animal species, strongly suggesting that coordination of chromosome capture, as described here, may be generally required. Animal oocytes store nutrients, hence the oocyte as well as its nucleus is exceptionally large (Lénárt and Ellenberg, 2003). To retain these stored nutrients for the fertilizable egg, meiotic divisions are extremely asymmetric, and thus the meiotic spindle is small and located near the cell cortex across animal species (Crowder et al., 2015). This specialized organization necessitates additional mechanisms to collect chromosomes scattered in the large nuclear volume, hence mechanisms to coordinate capture with these processes. Importantly, as the spindle assembly checkpoint is weak or inactive in oocytes, chromosome capture and its coordination is absolutely essential to produce euploid eggs, and thereby essential for sexual reproduction of the species. Intriguingly, actin structures around chromosomes have been reported at early steps of oocyte meiosis in jellyfish (Amiel and Houliston, 2009), tunicates (Prodon et al., 2006), *Xenopus laevis* (Weber et al., 2004; Yamagishi and Abe, 2018), as well as mouse (Mogessie and Schuh, 2017). It will be exciting to see in the future whether actin has similar, essential functions in coordinating chromosome capture in these species as well.

Additionally, related mechanisms may play a role in mitosis of somatic cells as well. Although completing search and capture as quickly as possible may intuitively appear the optimal solution, delaying chromosome capture and coordinating it with other cellular events is likely to provide advantages and may contribute to preventing chromosome loss and aneuploidy also in mitosis. For example, it has been shown that in somatic cells chromosomes are prearranged in a specific spatial configuration in prometaphase, before capture by microtubules to accelerate the spindle assembly (Magidson et al., 2011). This suggests that mechanisms may indeed exist also in somatic cells to delay capture of chromosomes until they are favorably positioned for rapid and efficient spindle assembly.

## Materials and methods

### Oocyte collection, maturation, and injection

Starfish (*Patiria miniata*) were obtained from Southern California (South Coast Bio-Marine LLC, Monterey Abalone Company, or Marinus Scientific Inc). Animals were maintained in seawater aquariums at 16°C at European Molecular Biology Laboratory’s Marine Facility. Oocytes were isolated, and mRNAs and other fluorescent markers were injected into the oocytes by using mercury-filled microneedles, as described previously (Jaffe and Terasaki, 2004; Borrego-Pinto et al., 2016b). mRNA was injected 24–48 h before to allow protein expression, whereas fluorescently labeled protein markers were injected a few hours before imaging. Oocytes were induced to enter meiosis by addition of 10 µM 1-methyladenine (1-MA; Acros Organics). NEBD normally initiated 25 min after 1-MA addition, and only those oocytes that started NEBD within 40 min were considered for analysis.

### Live-cell fluorescent markers

H2B-3mEGFP and H2B-mCherry, EB3-3mCherry, mEGFP-ArpC1, 3mEGFP-UtrCH, and 3mCherry-UtrCH were subcloned to the pGEM-HE vector (Borrego-Pinto et al., 2016b). mRNA was synthesized in vitro from the linearized DNA template by using the AmpliCap-Max T7 High Yield Message Maker kit (Cellscript), followed by polyA-tail elongation (A-Plus Poly(A) Polymerase Tailing kit; Cellscript). mRNAs were dissolved in water (typical concentration, 3–5 µg/µl) and injected into the oocytes up to 5% of the oocyte volume. Histone H1 and UtrCH (Burkel et al., 2007) were labeled with Alexa fluorophores as described previously (Bun et al., 2018). Phalloidin labeled with the indicated Alexa fluorophores (Invitrogen) was dissolved in methanol and was then air-dried before use and dissolved in PBS for microinjection and immunostaining. For phalloidin, microinjection was performed 2 min after NEBD into the nuclear area. H1–Alexa Fluor 568, UtrCH–Alexa Fluor 568, Arp2/3–Alexa Fluor 488 (gift from B. Bugyi, University of Pécs, Pécs, Hungary), and mEGFP-Ndc80 (gift from S. Maffini and A. Musacchio, Max-Planck Institute, Dortmund, Germany) proteins were injected into the oocytes before maturation. RanT24N and RanQ69L (gift from R. Walczak and I. Mattaj, European Molecular Biology Laboratory, Heidelberg, Germany; final concentration, ∼15 µM) protein was injected right before NEBD.

### Drug treatments

For all inhibitor treatments, oocytes were first transferred to an Ibidi dish (catalog number 80131). To acutely depolymerize F-actin, Latrunculin B (final concentration, 7 µM) was added directly on the microscope stage in the form of a double-concentrated solution to the equal volume of seawater contained in the chamber. Nocodazole (final concentration, 3.3 µM) and Cytochalasin D (final concentration, 40 µM) were diluted from DMSO stocks in seawater and added simultaneously with 1-MA. Ciliobrevin D (final concentration, 150 µM) was added 10 min before NEBD. Oocytes were incubated with CK-666 (final concentration, 0.5 mM) for 1 h before maturation. Zeocin (final concentration, 100 mg/ml) was added 3.5 h before maturation. In all cases, control oocytes were treated at the same times with the corresponding amount of the DMSO solvent.

### Immunostaining

Oocytes were fixed at desired times by the fixative composed of 100 mM Hepes, pH 7.0, 50 mM EGTA, 10 mM MgSO_4_, 0.5% Triton X-100, 1% formaldehyde, and 0.1% glutaraldehyde, as described by (Strickland et al., 2004). Samples were additionally treated with ImageIT (Thermo Fisher Scientific) to reduce unspecific antibody binding and mounted with the antifade agent ProLongGold (Thermo Fisher Scientific) between single layers of double-sided adhesive tape (Scotch). Microtubules were visualized by an α-tubulin antibody (1:400 DM1α; Sigma-Aldrich) and goat anti–mouse Alexa Fluor 488 or 568 secondary antibodies (1:500). mEGFP-ArpC1 signal was enhanced by an α-GFP antibody (1:400; ab6556; Abcam). Fixed oocytes were imaged according to Niquist criteria (pixel size, 38 nm; z-step, 130 nm).

### Image acquisition and processing

Live cell movies were acquired on a Leica SP5 confocal microscope by using a 40× HCX PL AP 1.10 NA water-immersion objective lens (Leica). Fixed samples were imaged on a Leica SP8 microscope equipped with the HC PL APO 1.40 NA 100× oil-immersion objective. Where indicated, images were deconvolved by using the Huygens software (Scientific Volume Imaging). Fast acquisition rate images were acquired on a Zeiss LSM 880 confocal microscope equipped with the AiryFast module and 40× C-Apochromat LD 1.1 NA water-immersion objective lens (typical settings to image a volume of 70 × 70 × 60 µm volume, 306 × 306 pixels [pixel size, 224 nm] in *xy*, and Z step of 1.4 µm, allowing a time resolution of one stack every 3 s). AiryFast pixel reassignment and deconvolution was performed in the Zeiss Zen Black software. Imaging was performed at controlled temperature (19–21°C).

Unless specified, images were loaded and adjusted for brightness and contrast, projected (maximum-intensity projection and/or temporal color code for time projections), and filtered (Gaussian blur, typically 0.5–1 pixels) in Fiji/ImageJ (Schindelin et al., 2012). Chromosome tracking was performed in 3D using either a custom Matlab (MathWorks) routine (Mori et al., 2011; Monnier et al., 2012) or Imaris (Bitplane). Chromosome capture events were identified manually by combining several quantitative measures and by examining the 3D trajectories, as well as plots of the chromosome–AP distance over time. Specifically, a chromosome-capture event was identified as a time point followed by at least four subsequent steps of unidirectional and fast motion (Fig. S1 B, chromosome 2), or at least two such steps if it coincided with a change in the overall direction (Fig. S1 B, chromosome 1). Every capture event was confirmed by examining the 3D trajectories (Fig. S1 B), as well as plots of the chromosome–AP distance over time (Fig. S1 C). These latter plots visualize the poleward/radial velocity component corresponding to the expected direction of transport on astral microtubules (Fig. S1 C). By these stringent criteria, we were able to identify capture events in ∼50% of chromosome trajectories in an unbiased manner (Fig. S1, E and F). To calculate the mEGFP-ArpC1 or 3mCherry-UtrCH intensity around chromosomes, we tracked chromosomes in Imaris and used a custom XTension script in Matlab to define a spherical volume around the chromosome and measured the intensity contained within. F-actin network intensity was quantified by measuring the mean fluorescent intensity in fixed size regions on individual confocal slices over time and normalized between minimum and maximum values. Analysis of tracks was performed, and plots were generated in Matlab. All figures were assembled in Adobe Illustrator CS6.

### Simulations

Chromosome congression was modeled in the Cytosim software (Nedelec and Foethke, 2007). The simulation was performed in a 3D spherical geometry 70 µm in diameter, corresponding to the mean size of the starfish oocyte nucleus and kinetochores matching in size and shape to experiments (Fig. S4 E). Microtubules were modeled as dynamic, nonflexible polymers nucleated from the centrosomes with a distance-dependent catastrophe rate (Fig. S4 D). Centrosomes were static and positioned 3 µm from the cell cortex and 6 µm apart from each other (Fig. S4 A). Microtubules touching the cell cortex underwent catastrophe immediately. No chromatin-mediated microtubule nucleation was observed experimentally or included in simulations (Fig. S4 F). The inactivation of kinetochores to prevent early capture events was modeled by setting the microtubule binding rate to 0 for the first 4 min after NEBD and then increasing it to 10, then 30, 70, and finally 100%, every minute, 4–8 min after NEBD.

As we focused here on chromosome capture, we did not represent the F-actin network by explicit filaments. Instead, we implemented a velocity field acting on the chromosomes to displace them as mediated by isotropic contraction of the F-actin network (Mori et al., 2011; Bun et al., 2018). The speed of any chromosome is constant in time, and it is proportional to its distance from the AP at NEBD onset (*t* = 0). Thus, at any time, the flow is directed toward a single point *p* at the AP. All chromosomes would converge to this point at the same time, *t_x_* = 15 min; the transport, however, stops at a time *t_m_* = 12.5 min. The resulting speed of a chromosome located at position *x* at time *t* is *v*(*x,t*) = (*p* − *x*)/(*t_x_* − *t*) for t ∈ [0, t_m_] and *v*(*x,t*) = 0 for *t* > *t_m_*. Another independent term representing the Brownian motion of a sphere of radius 0.8 µm embedded in a fluid of viscosity 0.1 Pa s is added to realistically reproduce chromosome motion. In the simulation, chromosome capture or transport by microtubules is not affected by the F-actin network velocity field, which is acting only on the chromosomes. A comparison of the chromosome-approach rate from the experimental data and in the model is shown on Fig. S4 C.

To decrease computational costs, the simulation time-step was set to 0.05 s, and microtubule rotation was neglected. Parameters of the model are tabulated in Fig. S4 B. The configuration file to run simulations is available on request. Cytosim is an Open Source project hosted on http://github.com/nedelec/cytosim.

### Online supplemental material

The supplemental material contains four figures showing details of the high-resolution tracking and identification of chromosome capture events (Fig. S1), details of the quantification of microtubule dynamics (Fig. S2 A), generation of the chromatin fragments by Zeocin (Fig. S2 B), as well as F-actin network stabilization (Fig. S2 C). The complete dataset for DMSO- and Latrunculin B–treated oocytes in Fig. 5 (E and F) is shown in Fig. S3. The details of the computer simulations are shown in Fig. S4. The supplemental material also includes six videos showing single events of chromosome capture (Video 1), F-actin disassembly by acute Latrunculin B treatment (Video 2), high temporal resolution chromosome tracking (Video 3), local collapse in the F-actin network caused by captured chromosomes (Video 4), and the kinetics of Arp2/3-nucleated patches on the chromosomes (Video 5). Video 6 shows the three simulations recapitulating chromosome capture kinetics.

## Supplementary Material

Supplemental Materials (PDF)

Video 1

Video 2

Video 3

Video 4

Video 5

Video 6
