# Supplementary material for: F-Actin nucleated on chromosomes coordinates their capture by microtubules in oocyte meiosis
Source: J Cell Biol. 2018 Aug 6;217(8):2661–74. doi: 10.1083/jcb.201802080 (PMC6080919; doi:10.1083/jcb.201802080)
Supplement: Supplemental Materials (PDF) [file JCB_201802080_sm.pdf]

Supplemental Material

Burdyniuk et al., https://doi.org/10.1083/jcb.201802080

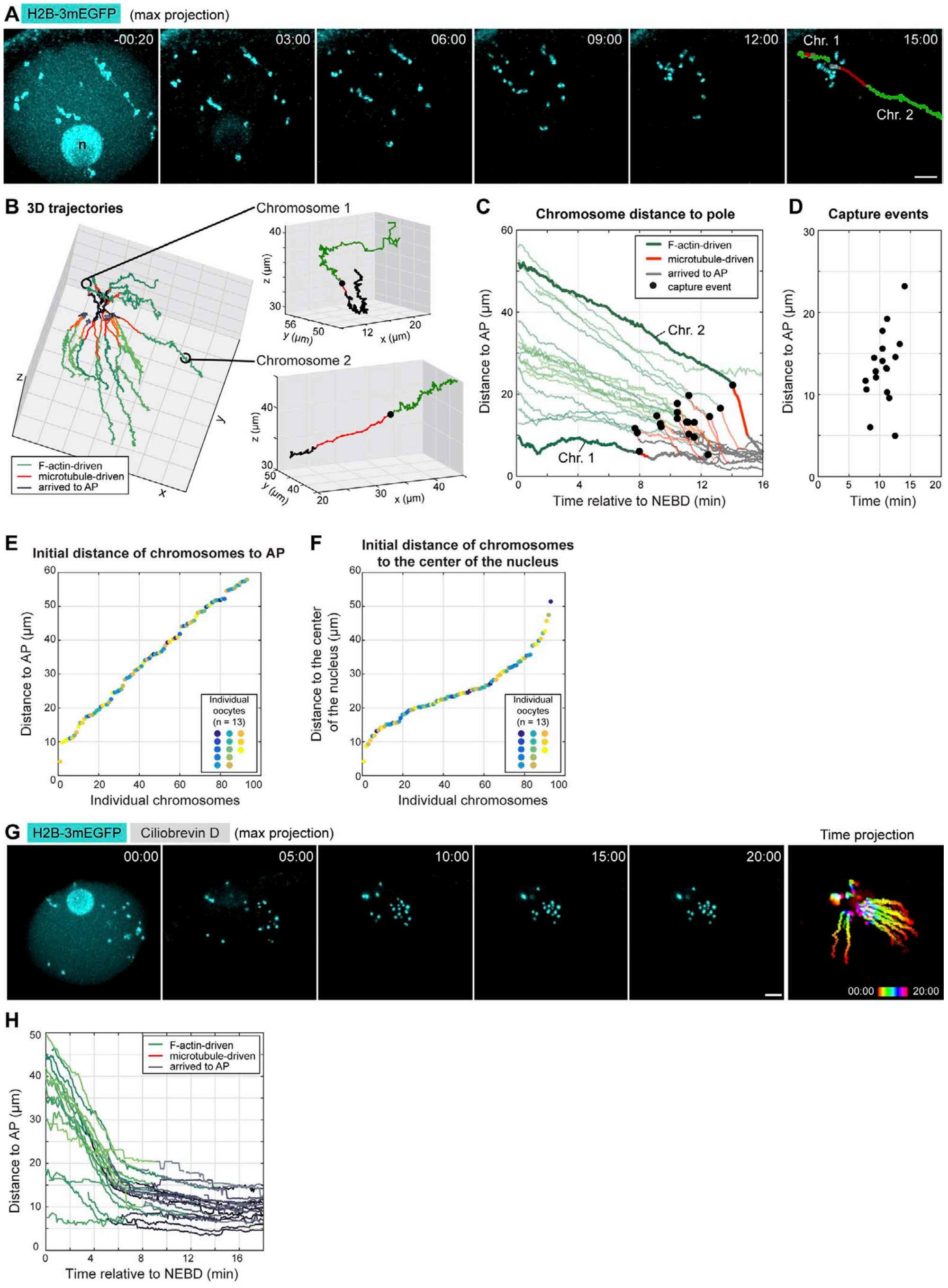

Figure S1. **Identification of the chromosome capture events and dynein-dependent chromosome transport. (A)** Selected maximum-intensity z-projections from a confocal time series though the oocyte's nuclear region during chromosome congression. Chromosomes (H2B-3mEGFP) are shown in cyan. n, nucleolus. **(B)** 3D plot of chromosome trajectories derived from the video shown in A, with trajectories of one of the farthest and one of the closest chromosomes shown separately. Black dots, capture events. **(C)** Plot of chromosome distance to the AP over time for the same dataset shown in A and labeled as on B. **(D)** Plot of capture events identified on C. **(E and F)** Distances from the AP and from the center of the nucleus at NEBD for individual chromosomes from the dataset shown in Fig. 2 B (DMSO) to illustrate homogeneous, unbiased sampling of chromosomes in the nuclear area. **(G)** Maximum-intensity z-projection from a time series of an oocyte expressing H2B-3mEGFP to label chromosomes. Ciliobrevin D was added 10 min before NEBD. Right: Temporal color-coded maximum projection. **(H)** Plot of chromosome distance to the AP over time in Ciliobrevin D–treated oocytes. Time is given as minutes:seconds relative to NEBD. Bars, 10 μm.

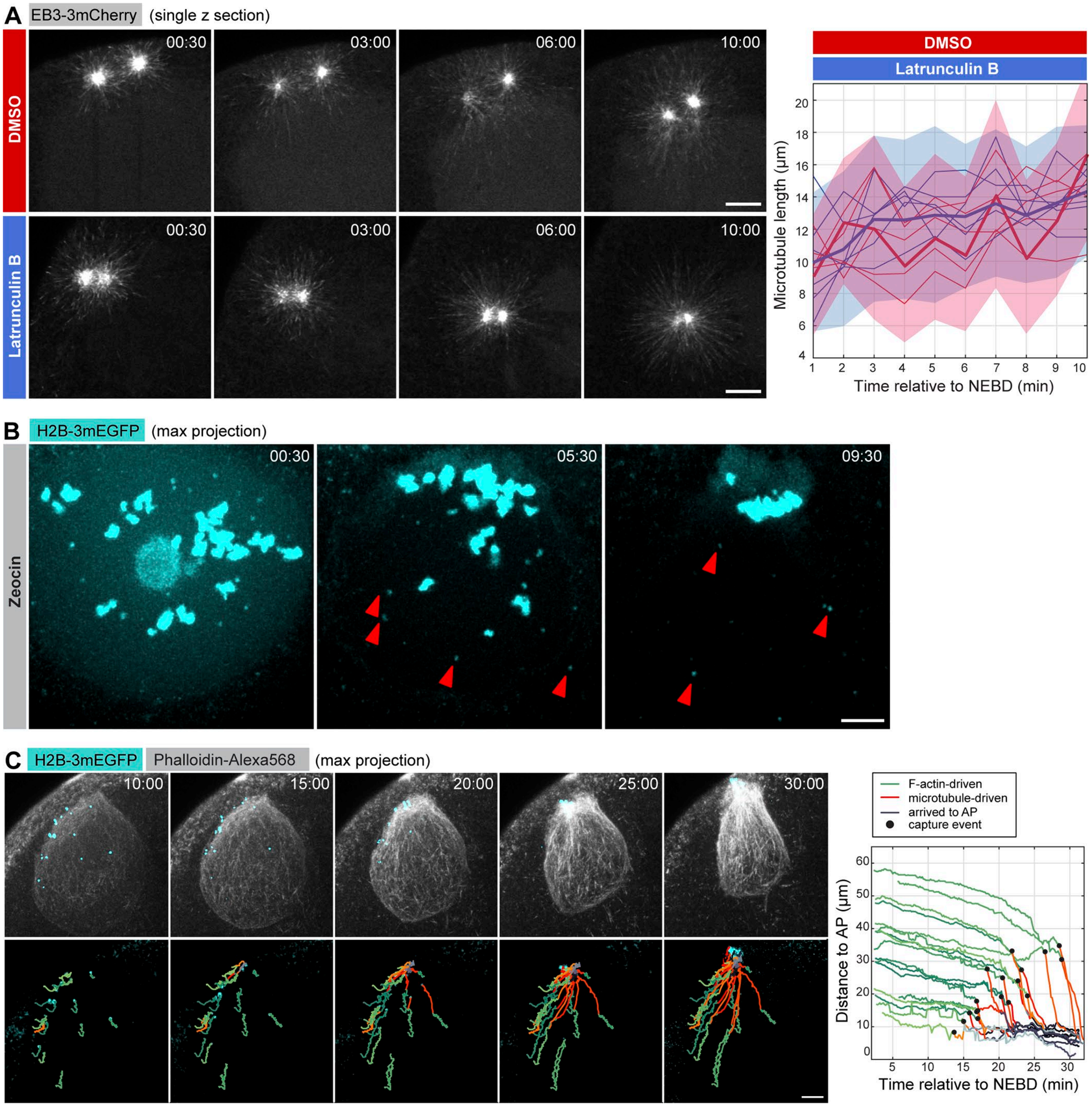

Figure S2. **F-actin network does not interfere with chromosome capture and transport. (A)** Single confocal slice from a time series of an oocyte expressing EB3-3mCherry to label microtubule plus-tips. Right: Histogram showing the microtubule length distribution in DMSO control (red) and Latrunculin B–treated (blue) oocytes. Thin lines, data from individual oocytes; thick lines, mean for six oocytes; shaded areas represent standard deviation. **(B)** Maximum-intensity z-projection from a time series of an oocyte expressing H2B-3mEGFP to label chromosomes. Arrowheads indicate chromatin fragments resulting from the treatment with the DNA-damaging agent, Zeocin. **(C)** Maximum-intensity z-projection from a time series of an oocyte expressing H2B-3mEGFP to label chromosomes. Two minutes after NEBD, the oocyte was injected with a pulse of Phalloidin–Alexa Fluor 568 next to the nuclear area to stabilize filaments of the F-actin network. Right: Plot of chromosome distance to the AP over time. Time is given relative to NEBD as minutes: seconds. Bars, 10 µm.

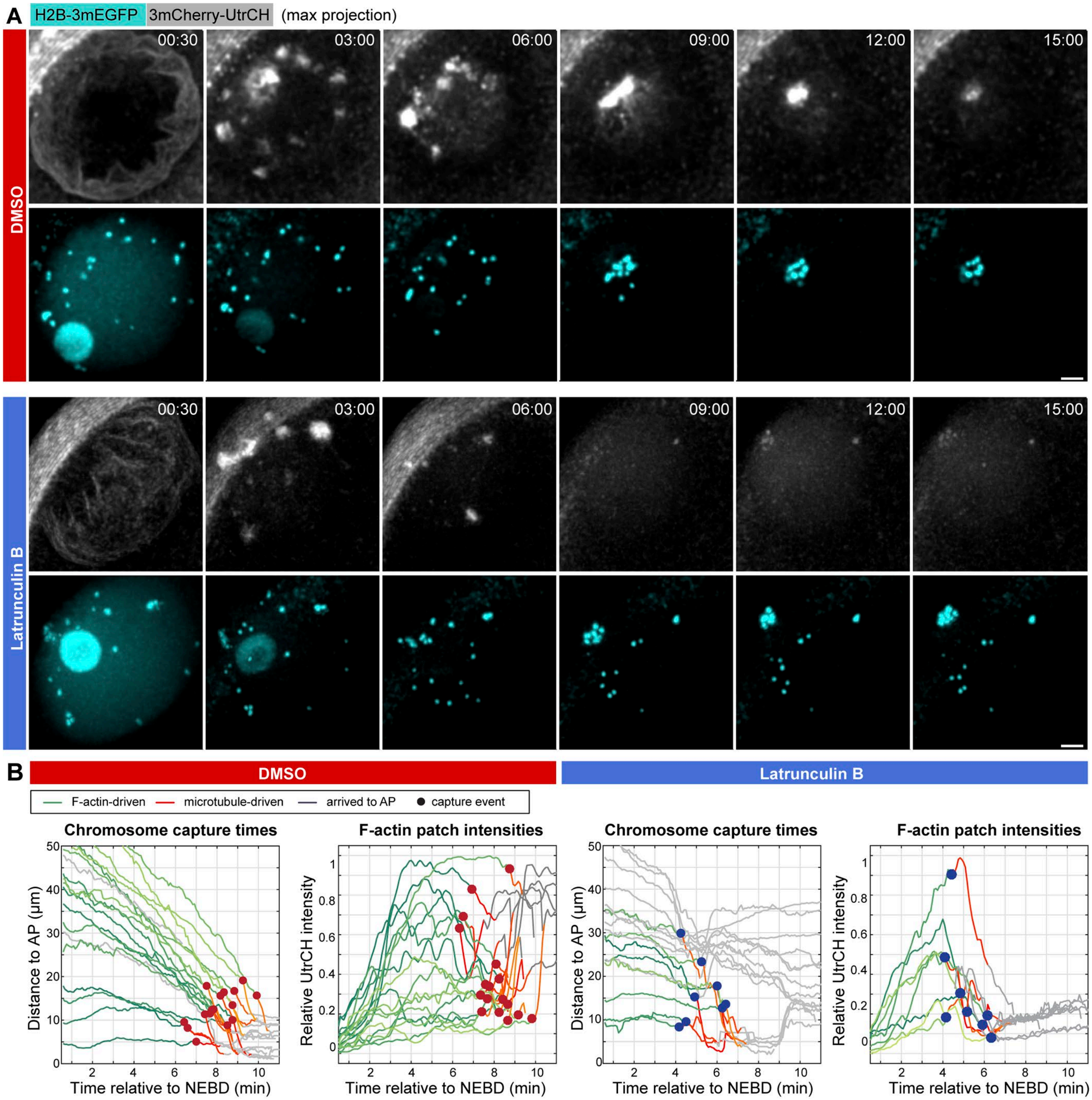

Figure S3. **Complete dataset for quantification of F-actin patch disassembly kinetics. (A)** Selected maximum-intensity z-projections from a 3D confocal time series though the oocyte's nuclear region during chromosome congression for control and Latrunculin B–treated oocytes shown in Fig. 5 (E and F). Chromosomes (H2B-3mEGFP) are in cyan and F-actin (3mCherry-UtrCH) in gray. Bars, 10 µm. **(B)** Left: Plot of chromosome distance to the AP over time for control and Latrunculin B–treated oocytes shown in A. Right: Normalized 3mCherry-UtrCH intensity profile for chromosomes tracked on the left. Intensity is calculated in a 5-µm-diameter sphere around the chromosome's center of mass. Time is given relative to NEBD as minutes:seconds.

A

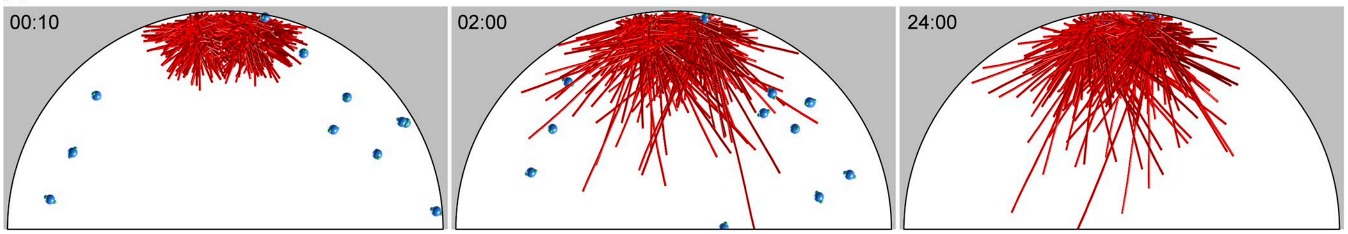

B

Parameter	Value	Source
Geometry (3D)		
diameter of the nucleus	70 µm	measured
number of chromosomes	22	measured
diameter of chromosomes	1.6 µm	measured
diameter of kinetochores	0.5 µm	measured
viscosity	0.1 Pa.s	(Bun et al., 2018)
Microtubule dynamics		
number MTs (/centrosome)	500	estimated
growth speed	0.5 µm/min	(Magidson et al., 2015)
shrinking speed	1 µm/min	(Magidson et al., 2015)
catastrophe rate*	0.15	estimated
rescue rate	0	(Magidson et al., 2015)
motor speed (~dynein)	-9.2 µm/min	measured
Simulation		
Simulation time step	0.05 s	optimized
MT segment length	0.25 µm	optimized

*distance dependent

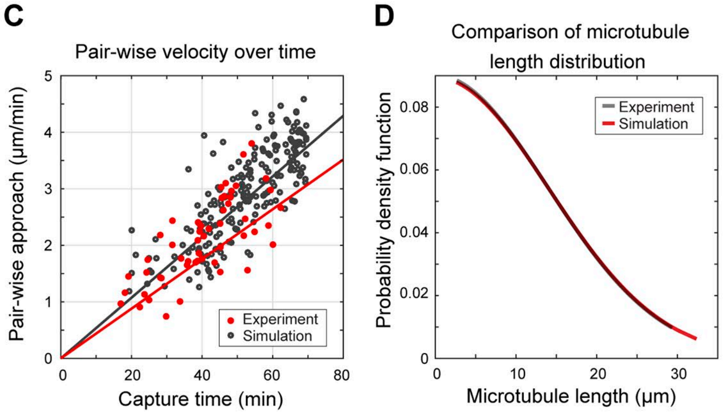

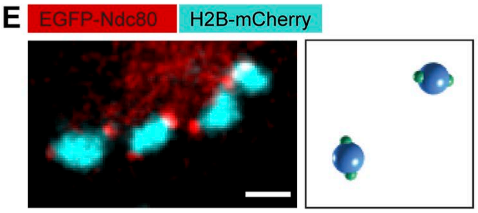

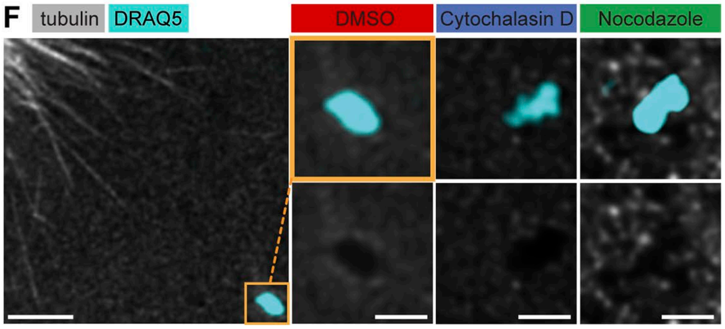

Figure S4. **Details of the computer simulations. (A)** Renderings of the 3D computer simulation in Cytosim: the nucleus is represented as a sphere with a diameter of 70 µm. Chromosomes (cyan) with two kinetochores each (green) are transported by the contractile F-actin network. After capture of kinetochores by microtubules, chromosomes are transported by dynein to the centrosomes. **(B)** Table summarizing the main parameters used in the model. **(C)** Chromosome pairwise velocity analysis to derive the network contraction rate, as described by Bun et al. (2018). Comparison between experimental data (red) and simulation (gray). **(D)** Microtubule length distribution in oocytes (red) and simulations (black) fitted with the half-normal distribution function. Microtubule length was compared at time 3 min to 10 min after NEBD, when asters reach full size. **(E)** Chromosome and kinetochore morphology in the experiment versus simulation. Left (experiment): Spindle area during prometaphase, in an oocyte expressing H2B-3mEGFP to label chromosomes and injected with mEGFP-Ndc80 protein to label kinetochores. Single deconvolved confocal slice. Bar, 2 µm. Right (simulation): Chromosomes are represented as 1.6-µm spheres (blue) each with two 0.5-µm kinetochores located on the opposite sides. **(F)** Chromatin-mediated microtubule nucleation is not active during the initial chromosome congression. Single deconvolved confocal slices through an oocyte fixed 8 min after NEBD and immunostained for tubulin (DM1α, gray), chromosomes (Draq5, cyan), and Phalloidin–Alexa Fluor 568 for F-actin (gray). Oocytes were treated before maturation with Cytochalasin D, Nocodazole, or DMSO as indicated. Bars: (main images) 5 µm; (smaller images) 2 µm.

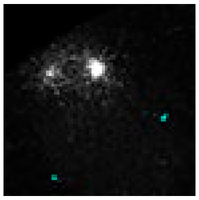

Video 1. **After F-actin–driven transport, chromosomes form lateral attachments and are transported along the microtubules to the AP.** Confocal sections taken every 0.7 s through the nuclear region of an oocyte expressing EB3-3mCherry to visualize microtubule plus-tips (gray) and H2B-3mEGFP to label the chromosomes (cyan). Video starts at 06:25 after NEBD and runs for 8 min. Imaged area: 51.5 × 51.5 µm. Selected frames are shown in Fig. 1 B.

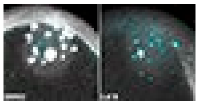

Video 2. **F-actin and chromosome dynamics upon acute F-actin depolymerization.** Maximum-intensity z-projections through the nuclear region of live oocytes expressing H2B-3mEGFP to label chromosomes and 3mCherry-UtrCH to label F-actin. Latrunculin B, or corresponding amount of DMSO, was added at NEBD onset (00:00). Video starts at 00:30 after NEBD and runs for 23 min. Time step: 5 s. Imaged area: 94 × 94 × 70.5 µm. Still frames from this dataset are shown in Fig. 2 B.

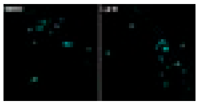

Video 3. **Chromosome capture is coordinated in F-actin–dependent manner.** Maximum-intensity z-projections through the nuclear region of live oocytes expressing H2B-3mEGFP to label chromosomes. Latrunculin B, or corresponding amount of DMSO, was added at NEBD onset (00:00). Video starts at 00:30 after NEBD and runs for 17 min. Time step: 3 s. Imaged area: 68 × 68 × 60 µm. Still frames from this dataset are shown in Fig. 2 C.

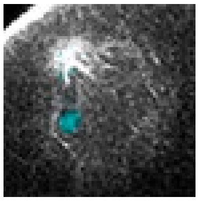

Video 4. **Chromosomes transported along microtubules are pulled through the F-actin network.** Single confocal sections acquired every 1 s of the nuclear area of an oocyte expressing 3mEGFP-UtrCH (gray) and H2B-mCherry (cyan). Video starts at 15:00 after NEBD and runs for 3 min and 24 s. Imaged area: 60 × 60 µm. Still frames from this dataset are shown in Fig. 3 C.

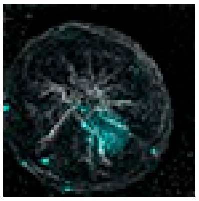

Video 5. **F-actin patch disassembly kinetics correlates with chromosome capture by microtubules.** Maximum-intensity z-projections of z-stacks acquired every 13 s through the nuclear region of live oocyte expressing mEGFP-ArpC1 (gray) and H2B-mCherry (cyan). Video starts at NEBD onset (00:00) and runs for 15 min. Imaged area: 82 × 82 × 62 µm. Still frames from this dataset are shown in Fig. 5 A.

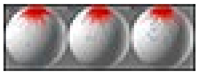

Video 6. **Simulation of microtubule search-and-capture in starfish oocytes.** Video starts at 00:00 at NEBD and runs for 15 min. Still frames from these datasets for all three simulation models are shown in Fig. 6 A. See the corresponding figure legend for details.

Reference

Bun, P., S. Dmitrieff, J.M. Belmonte, F.J. Nédélec, and P. Lénárt. 2018. A disassembly-driven mechanism explains F-actin-mediated chromosome transport in starfish oocytes. *eLife*. 7:e31469. https://doi.org/10.7554/eLife.31469
